# Chitin and Chitosan Nanofibers: Preparation and Chemical Modifications

**DOI:** 10.3390/molecules191118367

**Published:** 2014-11-11

**Authors:** Shinsuke Ifuku

**Affiliations:** Department of Chemistry and Biotechnology, Graduate School of Engineering, Tottori University, 4-101 Koyama-cho Minami, Tottori 680-8550, Japan; E-Mail: sifuku@chem.tottori-u.ac.jp; Tel.: +81-857-31-5592; Fax: +81-857-31-3190

**Keywords:** chitin, chitosan, nanofiber, chemical modification

## Abstract

Chitin nanofibers are prepared from the exoskeletons of crabs and prawns, squid pens and mushrooms by a simple mechanical treatment after a series of purification steps. The nanofibers have fine nanofiber networks with a uniform width of approximately 10 nm. The method used for chitin-nanofiber isolation is also successfully applied to the cell walls of mushrooms. Commercial chitin and chitosan powders are also easily converted into nanofibers by mechanical treatment, since these powders consist of nanofiber aggregates. Grinders and high-pressure waterjet systems are effective for disintegrating chitin into nanofibers. Acidic conditions are the key factor to facilitate mechanical fibrillation. Surface modification is an effective way to change the surface property and to endow nanofiber surface with other properties. Several modifications to the chitin NF surface are achieved, including acetylation, deacetylation, phthaloylation, naphthaloylation, maleylation, chlorination, TEMPO-mediated oxidation, and graft polymerization. Those derivatives and their properties are characterized.

## 1. Introduction

**Figure 1:**
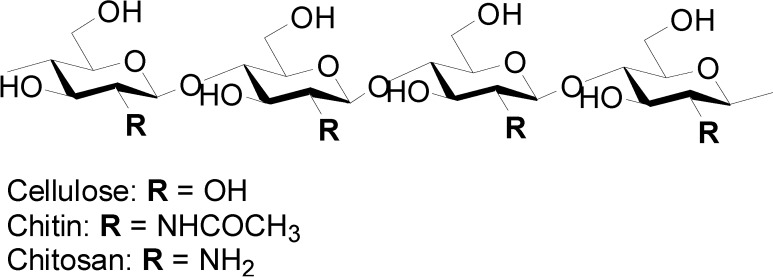
Chemical structures of cellulose, chitin, and chitosan.

A nanofiber (NF) is generally defined as a fiber of less than 100 nm diameter and an aspect ratio of more than 100 [1,2]. Properties of NF are quite different from those of microfibers, because NFs have a characteristic morphology, an extremely high surface-to-volume ratio [3], and unique optical [4] and mechanical properties [5], thus, it is important to develop a novel method of preparing NFs. The electro-spinning process is well known for producing artificial NFs from a wide range of polymers [6,7]. The process applies interactions between fluid dynamics, electrically charged surfaces, and electrically charged liquids. Recently, increasing interest has been paid to NFs from biopolymers, since these biopolymers are biodegradable, biocompatible, renewable, and sustainable. Nature produces a variety of bio-NFs, such as collagen triple helix fibers, fibroin fibrils, and keratin fibrils. These NFs basically form a complex hierarchical organization. This suggests that NFs can be extracted from these biomass-derived organizations by downsizing their structures. Among the variety of biopolymers, chitin and chitosan are known to be cellulose analogues with an *N*-acetylglycosamine repeating unit and the deacetylated derivative, respectively (Figure 1).

Chitin is the second most abundant biopolymer after cellulose, existing in the exoskeletons of crabs, prawns, and insects and the cell walls of fungi. Although chitin is produced in Nature at a rate of 10^10^ to 10^11^ tons per year, most chitin is thrown away as commercial waste [8]. This is obviously due to the low workability of the biopolymer, since commercially available chitin powder is not soluble in any solvent but precipitates immediately. Because of its linear structure, chitin has high crystallinity and is arranged as NFs. The NFs are basically embedded in a protein matrix. Since crab and prawn shells also have a complex hierarchical structure consisting of NFs [9], we expected that chitin NFs could be prepared by mechanical disintegration. The author developed a simple method for preparing chitin NFs by a top-down approach [10,11]. In this article, the author reviews the preparation of chitin NFs and chemical modifications to endow them with certain properties.

## 2. Preparation of Chitin and Chitosan Nanofibers 

### 2.1. Preparation of Chitin Nanofibers

#### 2.1.1. Nanofibers from Crab Shell [12]

**Figure 2:**
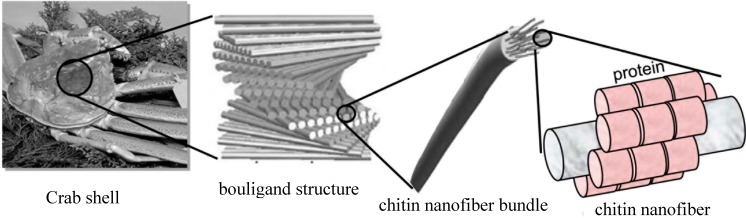
Schematic presentation of the exoskeleton structure of crab shell.

**Figure 3:**
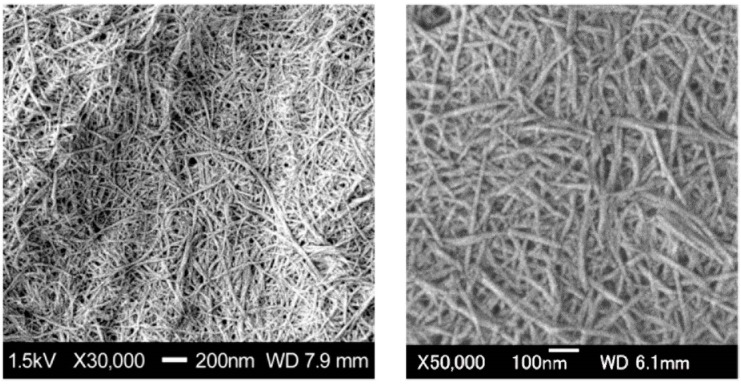
SEM images of chitin nanofibers from crab shell after grinder treatment.

Crab shells have a hierarchical organization with various structural levels (Figure 2). Chitin NFs were prepared from crab shells by a disintegration process [2]. To extract chitin NFs, the shells were first purified by a series of conventional chemical treatments and then subjected to mechanical treatment. Proteins and minerals were removed from the crab shells by aqueous NaOH and HCl treatments, respectively [13,14]. A grinder (Masuko Sangyo Co., Ltd., Kawaguchi, Japan) was efficient for disintegrating the chitin aggregates [15,16]. A pair of grinding stones effectively disintegrated the chitin organization. After one cycle of wet-type grinder treatment, the chitin slurry formed a gel, suggesting disintegration was accomplished due to a high surface-to-volume ratio. The chitin consisted of highly uniform NFs with a width of approximately 10 nm (Figure 3). The chitin NFs still had their original chemical and crystalline structures after a series of treatments. This simple but powerful method allows us to obtain homogeneous chitin NFs from waste crab shell in large amounts.

#### 2.1.2. Nanofibers from Prawn Shell [17]

The method of preparing chitin NF from crab shell is applicable to a variety of prawn shells, since prawn shell is also made up of a hierarchical organized structure. Three types of prawn shells were chosen as starting materials: *Penaeusmonodon* (black tiger prawn), *Marsupenaeus japonicas* (Japanese tiger prawn), and *Pandalus eous Makarov* (Alaskan pink shrimp). These species are widely cultivated around the world as important food sources, although their shells are often thrown away as food industrial waste. After proteins and minerals were removed, purified wet chitins extracted from the shells were disintegrated using a grinder. A uniform structure of the chitin NFs was observed. The fiber structure was very similar to that of NFs obtained from crab shells. Since the prawn exoskeleton is made up of a finer structure than crab shell, the mechanical disintegration of prawn shell was easier than that of crab shell [18,19].

#### 2.1.3. Nanofibers from Mushroom [20]

The cell walls of mushrooms also consist of chitin NFs, which form a complex with glucans [21,22]. Edible mushrooms are a source of dietary fiber, which is available as a food bulking or thickening agent, a film forming agent, and a stabilizer. We prepared chitin NFs from mushrooms referring the method described above. Five widely consumed species of mushrooms were selected: *Pleuotus eryngii* (king trumpet mushroom), *Agaricus bisporus* (common mushroom), *Lentinula edodes* (shiitake), *Grifola frondosa* (maitake), and *Hypsizygus marmoreus* (bunashimeji). Since the ingredients of mushroom cell walls are quite different from those of crab and prawn shells, a different method of extracting chitin from mushroom had to be used [23]. The chitin was disintegrated into chitin NF after grinder treatment. All NFs prepared from five different mushrooms were similar to those from crab and prawn shells. However, they formed a complex with several glucans on their surface. The content ratio of the glucan differed considerably depending on the species. The dietary nanosized fibers obtained from cultivable and edible mushrooms will have a wide range of applications a novel functional food ingredients.

#### 2.1.4. Nanofibers from Squid Pen e-Chitin [24,25]

Two types of chitin crystal are found in Nature: α- and β-chitins. Although α-chitin has an antiparallel mode, β-chitin has a parallel chain-packing mode. Most natural chitins have the α-type crystal structure, while the β-type chitin is present in squid pens. Fan and Isogai *et al.* prepared chitin NFs with a 3–4 nm width and a high aspect ratio from squid pen β-chitin. Mechanical treatment under acidic conditions is important for preparing chitin NFs. Cationic charges of the chitin NFs accelerate NF conversion. Moreover, lower crystallinity, the parallel chain-packing mode, and relatively weak intermolecular forces of squid pen β-chitin are also key factors in the preparation of chitin NFs.

#### 2.1.5. Nanofibers from Commercial Chitin Powder [26,27,28]

For the preparation of NF, extracted chitin must be kept wet to avoid strong inter-fibrillar coagulation. However, this is a disadvantage for the commercialization of chitin NF. The author developed a facile method of preparing chitin NFs from commercially dry chitin powder. Dry chitin was dispersed in acidic water and passed through a grinder. Although commercial chitin was made up of aggregates of NFs, the aggregates were easily converted into homogeneous NFs. This ease of conversion was attributed also to the electrostatic repulsion effect as described above. The repulsive force caused by the cationization of amino groups facilitated fibrillation into NFs. Several other organic acids can also accelerate disintegration. NFs from commercial chitin are advantageous because a large amount of chitin can be obtained within a few hours. A high-pressure waterjet system called the Star Burst instrument was also effective for the nanofibrillation of dry chitin. Chitin in aqueous acetic acid was passed through waterjet system equipped with a ball-collision chamber. The slurry was ejected from a small nozzle under high pressure. After mechanical treatments, the NFs became thinner as the number of treatments increased.

### 2.2. Preparation of Chitosan Nanofibers [29]

**Figure 4:**
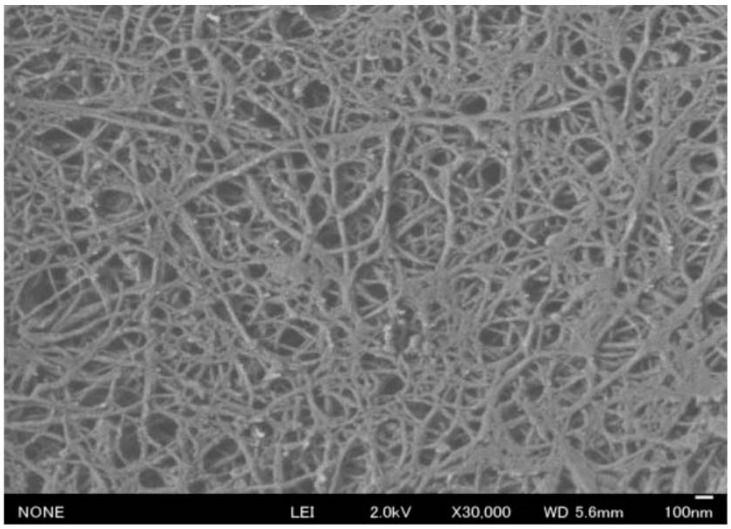
SEM image of chitosan nanofibers after high pressure waterjet treatment.

Chitosan is prepared by the deacetylation of chitin. Because of its biocompatibility and biodegradability as well as its cellular-binding, wound-healing, anti-bacterial, and anti-fungal properties, chitosan has great potential for many uses, including food, cosmetic, biomedical, and pharmaceutical applications [30,31,32,33,34]. The electro-spinning process is the typical method of artificially producing NFs from a polymer solution. However, the electro-spinning of chitosan is difficult due to the excessive surface tension of the chitosan solution [35]. If the characteristic NF structure of chitin is maintained after deacetylation, a downsizing process may be useful for the production of chitosan NF. Dry chitosan powder was treated by using the high-pressure waterjet system, and was disintegrated into NFs (Figure 4). 

However, the chitosan NFs became thinner as the number of treatment cycles increased. Here, in addition to chitin and cellulose NFs, chitosan NFs have been considered in bio-NFs.

## 3. Chemical Modifications of Chitin Nanofibers

### 3.1. Acetylation of Chitin Nanofibers [36]

**Scheme 1:**
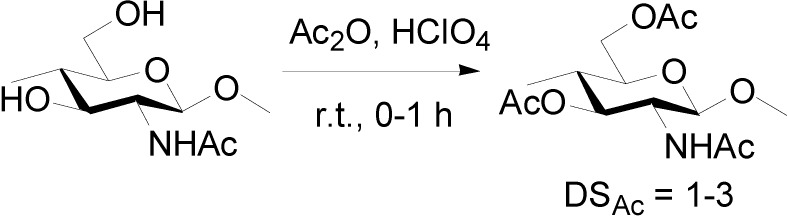
Acetylation of chitin nanofibers.

Surface modification by the introduction of certain functional group into hydrophilic hydroxyl groups is a promising method to expand the applications of chitin NFs. Modification using hydrophobic functional groups on NFs will improve dispersibility in nonpolar solvents, hygroscopicity, and adhesion properties with hydrophobic matrices in composite materials [37]. In a series of modifications, acetylation is a simple, popular, and inexpensive approach to modifying the surface properties [38,39,40]. Chitin NFs were acetylated in a mixture of acetic anhydride and perchloric acid at room temperature (Scheme 1).

The acetyl DS was controllable from 1 to 3 by adjusting the reaction time. Chitin NFs were acetylated completely within 1 h of reaction. The chitin NFs were acetylated heterogeneously from the surface to the core. First, the NF surfaces were immediately acetylated, and then the insides of the NFs were acetylated gradually. The NF structure was maintained even with a DS of 3, and the fiber thickness increased due to the bulky acetyl groups. Acetyl chitin NF/acrylic resin composites were prepared. Thanks to the size effect, all composite films were highly transparent, independent from acetyl DS. After 1 min acetylation, the moisture absorption of the nanocomposite film drastically decreased. Moreover, although the coefficient of thermal expansion of the acrylic resin used in this study was 6.4 × 10^−5^ °C^−1^, the thermal expansion of the composite film decreased to 2.3 × 10^−5^ °C^−1^. This is apparently due to the reinforcement effect of chitin NFs with low thermal expansion.

### 3.2. Preparation of Partially Deacetylated Chitin Nanowhiskers [41]

**Scheme 2:**
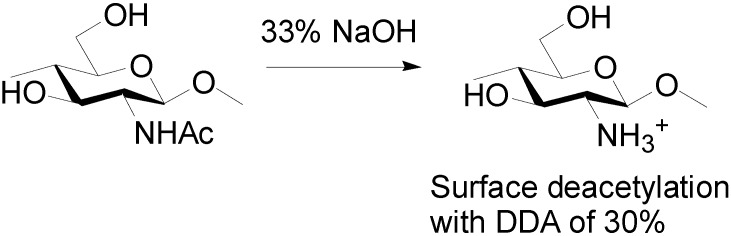
Partial deacetylation for chitin nanowhiskers.

Fan and Isogai *et al.* developed an efficient method to obtain chitin nanowhiskers by partial deacetylation (Scheme 2).

The acetamide group of chitin was converted into an amino group by deacetylation, and the electrically charged amino group on the chitin surface accelerated disintegration due to electrostatic repulsion. That is, α-chitin was partially deacetylated by 33% NaOH treatment. The degree of deacetylation was approximately 30%. The relative crystallinity and crystal size of the original α-chitin were maintained after deacetylation. This indicates that the deacetylation mainly occurred on the chitin surfaces. Transparent and highly viscous liquids were obtained by ultrasonic disintegration under acidic conditions. The liquids consisted of nanowhiskers. The average width and length were 6.2 nm and 250 nm, respectively. High cationic charges appeared on the chitin surfaces in high density in acidic water; this is significant for effective disintegration.

### 3.3. Surface Maleylation, Phthaloylation, and Naphthaloylation of Chitin Nanofibers [42,43]

**Scheme 3:**
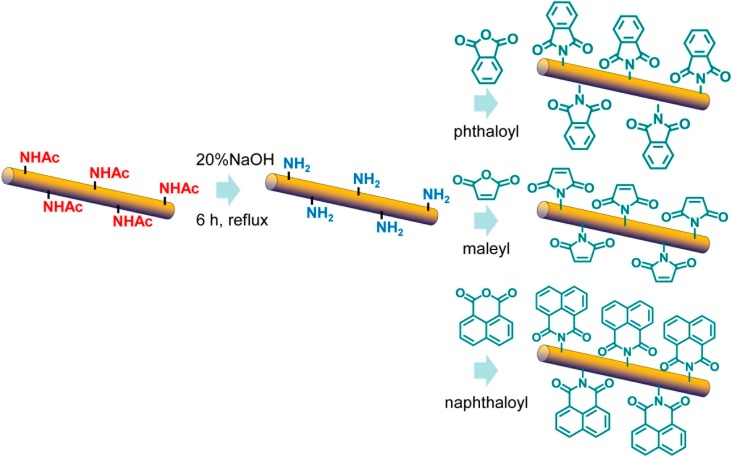
Phthaloylation, maleylation, and naphthaloylation of chitin nanofibers.

In chitosan chemistry, *N*-phthaloylation using phthalic anhydride is the most important protection reaction of the amino group [44]. Recently, highly chemoselective and quantitative *N*-phthaloylation of chitosan in aqueous media has been accomplished [45]. The reaction is useful for the surface modification of chitin NF, since NF disperses in water. Phthaloylation of chitin NF was achieved by reaction with phthalic anhydride in aqueous media (Scheme 3).

The functional group was quantitatively introduced into an amino group of the above described surface-deacetylated chitin NF. The NF network structure was maintained after phthaloylation. The NFs were dispersed homogeneously in organic solvents and even in hydrophobic aromatic solvents, due to the high solvation interactions with the phthaloyl group. The homogeneous dispersions in aromatic solvents had thermo-responsive properties and exhibited a dispersive-to-precipitate transition response at approximately 25 °C. Due to the presence of the phthaloyl group, the NF dispersion and the nanocomposite film completely eliminated harmful UV-B and -C light.

The surface modification technique was applicable to other modifications. Surface maleylation and naphthaloylation of chitin NF were achieved using maleic and naphthalic anhydride in water, respectively (Scheme 3). A maleyl or naphthaloyl group was sufficiently introduced to an amino group on a surface-deacetylated chitin NF. Each of these NFs also homogeneously dispersed in several organic solvents. The naphthaloyl NF dispersions in aromatic solvents also showed a dispersive-to-precipitate transition response. Moreover, the NF dispersion had exhibited UV-A adsorption in addition to UV-B and -C adsorption. The maleyl chitin NF formed a self-standing gel by free radical polymerization.

### 3.4. Preparation of Surface N-Halamine Chitin Nanofibers [46]

**Scheme 4:**
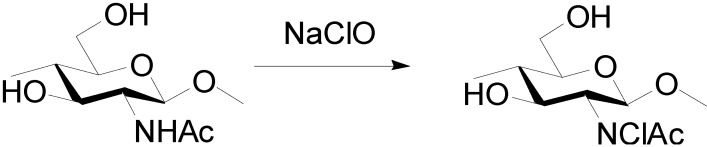
Preparation of surface *N*-halamine chitin nanofibers.

*N*-Halamines have attractive functions, such as their efficacy against microorganisms, stability, rechargeability, and nontoxicity to humans [47,48,49]. In halamines, chlorine has generally been used to connect with amine, amide, or imide groups due to the ease of modifying chlorine, its safety, its green reaction in water, and its low cost as a reagent. *N*-Halamine chitin NF film was prepared by the reaction of chitin NF film with sodium hypochlorite solution to endow the film with antibacterial and antifungal activities (Scheme 4).

The amount of active chlorine content introduced to the chitin NF film depended on the sodium hypochlorite concentration and the reaction time. The N-H bond was substituted to the N-Cl bond at the chitin NF surface. The nanochitin structure was maintained after the reaction. Although the active chlorine content of the film gradually decreased by disassociation of the N-Cl bond, chlorine was rechargeable into chitin NF by another treatment. The chlorinated chitin NF film showed strong efficacies against Gram-negative and -positive bacteria of *Escherichia coli* and *Staphylococcus aureus*, respectively. Moreover, the films showed 100% and 80% inhibition of spore germination when faced against *Alternaria alternata* and *Penicillium digitatum* fungi, respectively.

### 3.5. Chitin Nanocrystals Prepared by TEMPO-Mediated Oxidation [50]

**Scheme 5:**
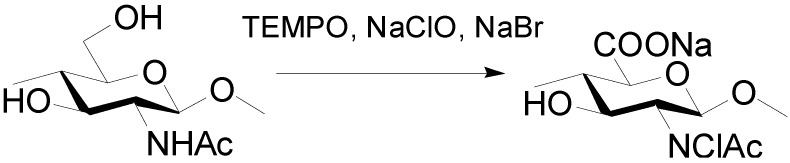
TEMPO-mediated oxidation for chitin nanocrystals.

Fan and Isogai *et al.* developed new methods for preparing chitin nanocrystals. Chitin nanocrystals dispersed in water were prepared by using 2,2,6,6-tetramethylpiperidine-1-oxyl radical (TEMPO)-mediated oxidation of α-chitin in water, followed by ultrasonic treatment (Scheme 5).

The TEMPO-oxidized chitin had a crystallinity as high as that of the original α-chitin, indicating the TEMPO-mediated oxidation occurred only at the chitin crystallite surfaces. *N*-deacetylation did not occur during the reaction. When the TEMPO-oxidized chitin was subjected to ultrasonic treatment in water, individualized chitin nanocrystals were obtained, with an average nanocrystal length and width of 340 and 8 nm, respectively.

### 3.6. Graft Polymerization of Acrylic Acid onto Chitin Nanofiber [51]

**Scheme 6:**
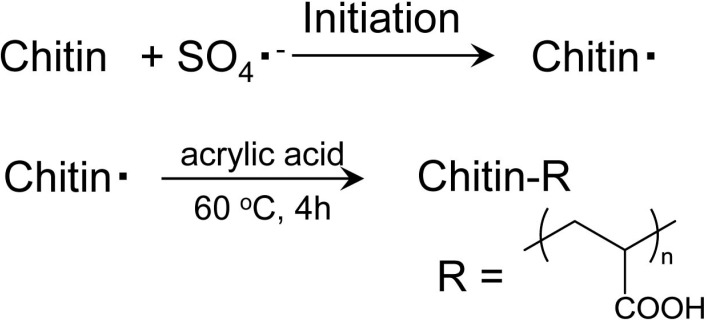
Graft co-polymerization of acrylic acid onto chitin nanofibers.

A few amino groups on the chitin fiber surface were cationized under acidic conditions, enabling the stable dispersion of NFs by electrostatic repulsion. On the other hand, chitin NFs precipitate in basic water. If anionic functional groups, such as carboxylate or sulfate groups, are introduced onto the chitin NF surface, the surface will have a negative charge under basic conditions, resulting in stable dispersion similar to that of TEMPO-oxidized chitin nanocrystals [50]. The grafting reaction was carried out with potassium persulfate as an initiator, which allows facile radical polymerization in aqueous media [52,53,54]. The process is based on a grafting-from process, where radicals are formed along the chitin polymer backbone followed by a free radical polymerization of acrylic acid monomer. Thus, graft copolymerization of acrylic acid (AA) on chitin NFs was carried out with potassium persulfate as a free radical initiator in an aqueous medium to endow the NF surface with a negative charge (Scheme 6).

The molar ratio of grafted AA increased with the AA concentration. The chitin NF structure was maintained after graft polymerization. Chitin NFs grafted with AA were efficiently dissociated and dispersed homogeneously in basic water because of the electrostatic repulsion effect between NFs. AA was grafted onto the surface and onto an amorphous part of chitin NFs, and the original crystalline structure of α-chitin was maintained. The thermal stability of the graft copolymer improved with the grafted AA content.

## 4. Conclusions

Chitin NFs were prepared from crab shells, prawn shells, squid pen, and mushrooms by a series of extraction treatments followed by mechanical treatment using a grinder or a high-pressure waterjet disintegration system. The obtained chitin NFs formed a fine NF structure. Although conventional chitin is insoluble and precipitates in water, chitin NFs can disperse homogeneously, so the dispersion is easy to handle and to shape into desired forms depending on the application [55,56,57]. Commercial chitin and chitosan powders are also easily converted into NFs by mechanical treatment, since these powders consist of NF aggregates.

Several chemical modifications of chitin NF surfaces were achieved: acetylation [36], deacetylation [41], phthaloylation [42], naphthaloylation [43], maleylation [43], chlorination [46], TEMPO-mediated oxidation [50], and graft polymerization [51]. Modification is a promising and effective way to design functional materials. A hydroxyl group is available to obtain chitin NF derivatives. Moreover, partially deacetylated chitin NFs have highly reactive amino groups. This enables facile surface modifications. Application of a reactive amino group for modification is important in order to define the separate roles of chitin and cellulose.

Chitin NFs have nanomorphologies and efficient mechanical properties owing to their extended crystalline structures. By making use of these characteristics, optically transparent chitin NF composites with acrylic resins were fabricated [58,59,60,61,62,63]. By virtue of chitin NFs’ reinforcing effect, chitin NFs could significantly increase the Young’s moduli and tensile strengths of acrylic resins, and decrease thermal expansion without losing transparency or flexibility. Moreover, chitin NFs have powerful biological activities and several applications were proposed in biomedical field [64,65,66,67,68]. For instance, they have anti-inflammatory actions via the suppression of NF-κB and MCP-1 activations and they suppress fibrosis in an acute ulcerative colitis mouse model, indicating that chitin NF is potentially a novel medicine or functional food for patients with inflammatory bowel disease [69,70]. Furthermore, the application of chitin NF to skin improved the epithelial granular layer, increased granular density, and resulted in lower production of TGF-β, indicating that chitin NFs can be incorporated into the manufacture of cosmetics or textiles [71,72]. Consequently, we expect that chitin NF with the above-mentioned properties will be widely used as a novel bio-material in the near future.
